# Alcohol Induced Mitochondrial Oxidative Stress and Alveolar Macrophage Dysfunction

**DOI:** 10.1155/2014/371593

**Published:** 2014-02-19

**Authors:** Yan Liang, Frank L. Harris, Lou Ann S. Brown

**Affiliations:** ^1^Department of Pediatrics, Emory University, Atlanta, GA 30322, USA; ^2^Children's Healthcare of Atlanta Center for Developmental Lung Biology, Atlanta, GA 30322, USA; ^3^Division of Pulmonary, Allergy & Critical Care Medicine, Emory University School of Medicine, Atlanta, GA 30322, USA; ^4^Division of Neonatal-Perinatal Medicine, Department of Pediatrics, Emory University, 2015 Uppergate Drive, Atlanta, GA 30322, USA

## Abstract

An alcohol use disorder increases the risk of invasive and antimicrobial resistant community-acquired pneumonia and tuberculosis. Since the alveolar macrophage (AM) orchestrates the immune response in the alveolar space, understanding the underlying mechanisms by which alcohol suppresses AM phagocytosis is critical to improving clinical outcomes. In the alveolar space, chronic alcohol ingestion causes severe oxidative stress and depletes antioxidants which are critical for AM function. The mitochondrion is important in maintaining cellular redox balance and providing the ATP critical for phagocytosis. The focus of this study was to understand how alcohol triggers mitochondrial reactive oxygen species (ROS), stimulates cellular oxidative stress, and induces AM dysfunction. The current study also investigated the capacity of the mitochondrial targeted antioxidant, mitoTEMPOL (mitoT), in modulating mitochondrial oxidative stress, and AM dysfunction. Using *in vitro* ethanol exposure and AMs from ethanol-fed mice, ethanol promoted mitochondrial dysfunction including increased mitochondrial ROS, decreased mitochondrial membrane potential, and decreased ATP. Treatment with mitoT reversed these effects. Ethanol-induced decreases in phagocytosis and cell viability were also attenuated with mitoT. Therefore, antioxidants targeted to the mitochondria have the potential to ameliorate ethanol-induced mitochondrial oxidative stress and subsequent decreases in AM phagocytosis and cell viability.

## 1. Introduction

Both acute and chronic alcohol consumption have well-documented effects on the immune system leading to increased susceptibility to community acquired pneumonia and tuberculosis [1]. When subjects with an alcohol use disorder get pneumonia, they are more likely to be infected with serious Gram-negative bacteria [2] and these increased risks occur even in those who do not meet the diagnostic criteria for an alcohol use disorder [3]. This results in a higher rate of intensive care use, longer inpatient stays, higher healthcare costs, and a 2–4 times greater mortality rate [4]. There is also an increased risk of ventilator-associated pneumonia which worsens the morbidity and mortality rates [5]. Alcohol abuse is also associated with a 2-3-fold increased risk of the acute respiratory syndrome (ARDS), representing ~50% of all ARDS cases with an average age of 30–35 [3]. For subjects without a history of alcohol abuse, pneumonia will lead to sepsis in ~35% of the cases and ~30% will progress to ARDS. In contrast, pneumonia will lead to sepsis in ~60% of the cases if the subject has a history of alcohol abuse and 70% will progress to ARDS [3].

A seminal feature is that chronic alcohol abuse causes severe oxidative stress in the fluid lining the alveolar space, which includes the depletion of the critical antioxidant glutathione (GSH) and oxidation of the GSH/GSSG redox state by ~40 mV in subjects with an alcohol use disorder [6, 7]. GSH depletion and oxidation within the alveolar space are particularly critical for alveolar macrophages (AM) since they are constantly bathed by this fluid and depend on this GSH pool for cellular uptake and protection against the oxidative stress generated during immune responses. Residing at the inner epithelial surfaces of airway and alveoli, AMs are the only lung phagocytes exposed directly to the environment. Therefore, AMs represent the first line of cellular defense in the lower respiratory tract [8]. However, oxidative stress can impair AM phagocytosis [9, 10]. In addition to impaired clearance of microbes, impaired phagocytosis can cause insufficient clearance of dying or dead cells and lead to pathological inflammation. Therefore, alcohol-induced oxidative stress can be a critical contributor to pulmonary pathophysiology, risk of infection, and contribute to the increased risk of tissue injury associated with ARDS.

There are multiple cellular sources of reactive oxygen species (ROS) including the mitochondria, the cytochrome P450 family, xanthine oxidoreductase, peroxisomes, cyclooxygenases, lipoxygenases, and the family of NADPH oxidases [11]. The consequences of the ROS depend on the type of the ROS generated, the amount of ROS, and where it is generated. Under resting conditions, the majority of the cellular ROS generated is derived from the mitochondria where ~90% of the oxygen used by a cell is consumed during energy metabolism [12]. In this mitochondrial process, nicotinamide adenine dinucleotide (NADH) is oxidized to support electrochemical coupling of oxidative phosphorylation and ATP synthesis [13–16]. However, respiration also generates ROS such as superoxide anions (O_2_^•−^), hydrogen peroxide (H_2_O_2_), and hydroxyl radicals (^•^OH). To protect against the ROS generated during respiration, mitochondria also maintain redox balance through numerous ROS defense systems including mitochondrial manganese superoxide dismutase (MnSOD), GSH, thioredoxin 2 (Trx2), and catalase [17]. Neutralization of mitochondrial ROS is critical for mitochondrial function and, ultimately, cellular functions but low-level concentrations of ROS are also required for signal transduction [18]. During respiration, the NADH is oxidized to NAD^+^ and the NAD^+^/NADH ratio has been recognized as a key regulator in energy metabolism, aging, and immunological functions [19]. For example, decreases in NAD^+^ or in the NAD^+^/NADH are associated with increased production of superoxide by the mitochondria and subsequent alteration of the mitochondrial redox system [20–22].

Alcohol metabolism can interrupt this complex integrated redox system within the mitochondria. Whether it is metabolized by alcohol dehydrogenase or cytochrome P450, the primary metabolite produced during alcohol metabolism is acetaldehyde. Within the mitochondria, acetaldehyde is metabolized by mitochondrial aldehyde dehydrogenase (ALDH2) [23] which uses NAD^+^ as a cofactor. Thus, acetaldehyde metabolism decreases mitochondrial NAD^+^ pools and increases NADH. The resulting decreases in the NAD^+^/NADH ratio and subsequent increases in mitochondrial ROS can change the mitochondrial redox balance leading to cellular oxidative stress and damage. Our research team has previously demonstrated that chronic alcohol ingestion resulted in impaired phagocytosis by AMs and chronic oxidative stress was central to the impaired immune functions [9, 10], while alcohol-induced upregulation of ROS through NADPH oxidases is linked to impaired phagocytosis; we speculated that there was also a role for alcohol-induced mitochondrial ROS generation in impaired AM phagocytosis. The results presented in this paper demonstrate that chronic alcohol ingestion in a mouse model induced mitochondrial ROS generation and mitochondrial dysfunction which contribute to impaired AM phagocytosis. Treatment with mitochondrial specific antioxidants reversed mitochondrial dysfunction and restored phagocytosis.

## 2. Materials and Methods

### 2.1. Mouse Model of Chronic Ethanol Ingestion

All animal studies were performed in accordance with the National Institutes of Health guideline outlined in the Guide for the Care and Use of Laboratory Animals. All described protocols were reviewed and approved by the Emory University Institutional Animal Care and Use Committee. Mice (C57BL/6; age 6–8 weeks) were fed standard laboratory chow *ad libitum* with incremental increases of ethanol in the drinking water over 3 weeks (5%/week) to a final concentration of 20%. Mice were maintained at 20% ethanol (EtOH) in the drinking water for 10–12 weeks (*n* = 5/group) [24, 25]. The controls were pair-fed in order to control for the calories due to EtOH as well as any differences in food intake. The weight of the chow consumed by the mice with ethanol in the drinking water is routinely determined and this historical data was then used to establish a pair-feeding model for the controls. This regimen produced clinically relevant elevations in blood alcohol concentrations of 0.12% ± 0.03, as published by our group [26] and others [27, 28]. After euthanasia, tracheas were cannulated and a bronchoalveolar lavage (BAL; three 1 mL of saline) performed. Mouse AMs (mAMs) were then isolated from the fluid by centrifugation at 1000 ×g for 10 min. After differential staining with Diff-Quik (Dade Behring, Newark, DE) and counting with a hemocytometer, the cell population was determined to be ~95% alveolar macrophages. The cell pellet was resuspended in RPMI 1640 medium containing 2% FBS and 1% penicillin/streptomycin and cells were incubated at 37°C in 5% CO_2_ atmosphere before the experiments outlined below were performed.

### 2.2. MH-S Cell Culture and EtOH Exposure

The mouse AM cell line, MH-S (American Type Culture Collection, Manassas, VA), was used as a model system for studying the direct effects of EtOH exposure *in vitro*. Cells were cultured in RPMI 1640 medium containing 10% FBS and 1% penicillin/streptomycin and incubated at 37°C in a 5% CO_2_ atmosphere. MH-S cells were treated with 0.2% EtOH for 5 consecutive days with the media changed daily. This EtOH concentration (0.2%) is representative of the blood alcohol content (BAC) when a 120 lb person consumes 5 drinks at a single sitting [28–30]. During the last 24 hr of the 5d EtOH treatment, some cells were also treated with the mitochondria-targeted antioxidant mitoTEMPOL (mitoT, 100 *μ*M) [31].

### 2.3. Measurement of Intracellular ROS Generation

After EtOH exposure, the cellular ROS sensitive probe CM-H_2_DCFDA (Invitrogen; Carlsbad, CA) and the mitochondrial superoxide probe mitoSOX (Invitrogen; Carlsbad, CA) were added to the medium (10 *μ*M, 30 min, 37°C). Cells were then harvested, washed, and resuspended in phosphate buffered saline (PBS) for FACS analysis by BD Canto II Flow Cytometer (Becton Dickinson, Franklin Lakes, NJ). CM-H_2_DCFDA and MitoSOX were excited at 488 nm and detected at 530 ± 15 nm or 585 ± 42 nm, respectively. Data analysis was performed using Flowjo (http://www.flowjo.com/).

### 2.4. Measurement of Mitochondrial Membrane Potential

After the different treatments, the mitochondrial membrane potential was determined by incubating the cells with tetra-methylrhodamine, ethyl ester (10 nM, 30 min, 37°C; TMRE; Sigma, St. Louis, MO). This cell-permeable, positively charged, red-orange fluorescent dye is readily sequestered by active mitochondria due to the relative negative charge of the fluorophore. However, depolarization of the mitochondrial membrane results in a failure to sequester TMRE. Cells were then harvested, washed, resuspended in PBS, and analyzed by FACS analysis. Data analysis was performed using Flowjo.

### 2.5. Measurement of Mitochondrial ATP Production

ATP production was measured by a plate reader bioluminescence assay following the manufacturer's instructions (abcam, Boston, MA). In brief, MH-S cells were harvested after the appropriate exposures, stained with Diff-Quik (Dade Behring; Newark, DE), and counted using a hemocytometer. 10 *μ*L of a cell resuspension (10^3^-10^4^ cells) was mixed with 100 *μ*L of the reaction mix for 5–10 min and then read in a luminometer. The ATP values were normalized to the cell count for each sample.

### 2.6. Colorimetric Assay for Measuring the Mitochondrial Ratio of NAD/NADH

EnzyChrom NAD^+^/NADH assay kit (Bioassay Systems; Hayward, CA) was used to determine the mitochondrial ratio of NAD^+^/NADH. In brief, mitochondria were isolated from MH-S cells or mAMs using a Mitochondria Isolation Kit (Thermo Fisher Scientific, Rockford, IL). NAD^+^ and NADH were then extracted with the extraction buffer provided in the assay kit, mixed with assay buffer, and absorbance-read at 565 nm.

### 2.7. Measurement of Phagocytosis and Cell Viability

To determine the phagocytic capacity of macrophages, pHrodo Red *S. aureus* bioparticles conjugate (Invitrogen, Carlsbad, CA) was added to the culture media according to the manufacturer's recommendations with ~2 × 10^6^ cells per 2 mg vial of pHrodo-labeled bioparticles. Cells were incubated with the pHrodo labeled bioparticles for 2 hrs and then collected for FACS analysis and data analysis by Flowjo. This phagocytosis assay is based on the fact that there is a minimal fluorescence signal when the pHrodo Red *S. aureus* bioparticle conjugate is adherent to the outer surface of the phagocyte. Once the *S. aureus* is internalized and incorporated into the acidic environment of the phagosome, the bioparticle conjugates emit a strong red fluorescence. Internalization was verified by live cell confocal imaging (Olympus FluoView FV1000, Center Valley, PA). To assess changes in viability due to ethanol, MH-S cells were stained with the Dead Cell Apoptosis Kit with Annexin V Alexa Fluor 488 and Propidium Iodide (PI) (Invitrogen, Carlsbad, CA) before analysis by flow cytometry.

### 2.8. Fluorescence Microscopy and Image Analysis

mAMs isolated from EtOH-fed and control mice were cultured overnight in 8-well cover glass bottom chambers (Lab-Tek; Scotts Valley, CA) with RPMI 1640 medium containing 2% FBS and 1% penicillin/streptomycin. Some mAMs were also treated with 500 *μ*M mitoT for 24 hrs. ROS probes CM-H_2_DCFDA (10 *μ*M) or mitoSOX (10 *μ*M) was added to the media and images were taken after a 30 min incubation. Images were acquired with Olympus FluoView FV1000 Confocal Microscope using a 63 × oil objective. Images were viewed and analyzed by FV10-ASW 2.0 (Olympus, Center Valley, PA). For mitochondrial morphology analysis, acquired images were subjected to particle analysis using ImageJ Particle Analyzer (National Institutes of Health (http://rsbweb.nih.gov/ij/)). After thresholding, individual particles (mitochondria) were analyzed for area, perimeter, circularity (4*π* × Area/(perimeter^2^)), and the lengths of major and minor axes of fit ellipse. From these values, form factor (FF; the reciprocal of circularity value) and aspect ratio (AR; major/minor) were calculated. Both FF and AR have a minimal value of 1 when a particle is a perfect circle and the values increase as the noncircle features of the particle increase. Specifically, AR is a measure of mitochondrial length and the increase of FF represents the increase of mitochondrial length and branching. This procedure is similar to mitochondrial morphology analysis as previously described [30, 32].

## 3. Results

### 3.1. Chronic EtOH Exposure Induced Mitochondrial ROS Generation

**Figure 1:**
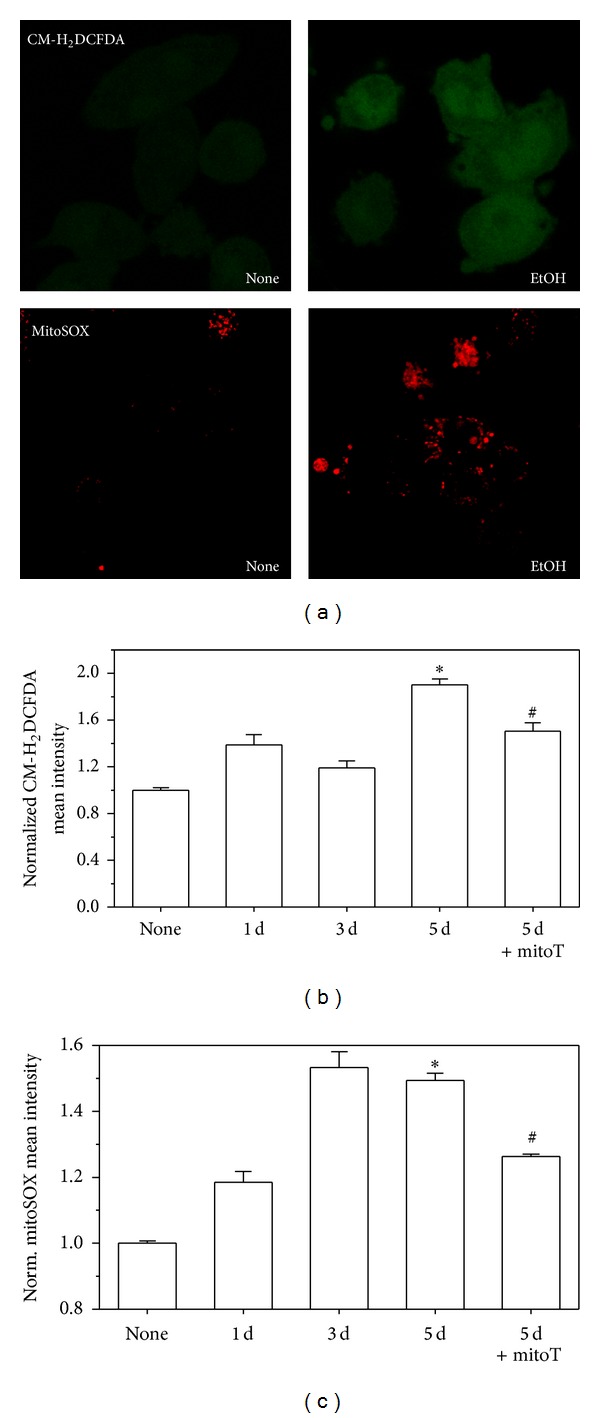
EtOH-induced ROS production in MH-S cells. Cultured MH-S cells were either untreated (none) or EtOH treated for 1d, 3d, and 5d (0.2%, *n* = 3). During the last 24 hr of EtOH treatment, some cells were cotreated with mitoT. The ROS probes CM-H_2_DCFDA for cellular ROS and mitoSOX for mitochondrial superoxide were added to the cell medium (30 min; 37°C). Cells were then harvested, washed, and resuspended in PBS for confocal microscopy (a) or FACS analysis ((b) and (c)). All values are expressed as mean ± SD and normalized to untreated conditions. *P < 0.05 for EtOH *versus* none and ^#^ *P* < 0.05 for 5d EtOH *versus* 5d of EtOH + mitoT.

**Figure 2:**
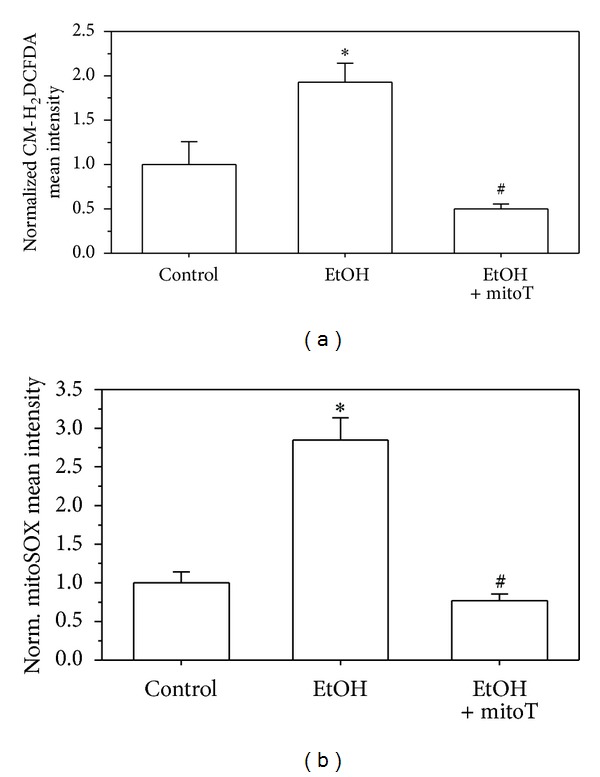
Chronic EtOH ingestion induced ROS production in mAMs. mAMs were collected from control and EtOH-fed mice (*n* = 4). The collected mAMs were cultured and treated with mitoT for 24 hrs. Cells were stained with CM-H_2_DCFDA (a) for cellular ROS and mitoSOX (b) for mitochondrial superoxide. Computer analysis of confocal microscopic images was used to quantify ROS generation. All values are expressed as mean ± SE and normalized to control conditions. *P < 0.05 for EtOH *versus* control and ^#^ *P* < 0.05 for 5d EtOH *versus* EtOH + mitoT.

CM-H_2_DCFDA is oxidized to DCF (dichloro-fluorescein) by cellular ROS [33] and ethanol increased DCF fluorescence (images in Figure 1(a)). MitoSOX Red, which selectively targets mitochondria and is rapidly oxidized by superoxide, was used to monitor mitochondrial superoxide production (images in Figure 1(a)). In MH-S cells, five days of EtOH exposure increased both cellular ROS and mitochondrial superoxide production by ~100% and 50%, respectively (Figures 1(b) and 1(c)). However, EtOH-induced upregulation of cellular ROS and mitochondrial superoxide in MH-S cells were reversed by treatment with the mitochondrial targeted antioxidant, mitoT. To determine whether chronic EtOH ingestion induced ROS generation *in vivo*, mAMs were isolated from control or EtOH-fed mice and stained with MitoSOX and CM-H_2_DCFDA before flow cytometry or confocal imaging. Chronic ethanol ingestion upregulated cellular ROS in mAMs by ~2-fold and mitochondrial superoxide by ~3-fold (Figures 2(a) and 2(b)). Similar to that observed with MH-S cells, 24 h *in vitro* treatments of the mAMs with mitoT reversed EtOH-induced cellular and mitochondrial ROS production.

### 3.2. Chronic EtOH Exposure Resulted in Mitochondrial Dysfunction

**Figure 3:**
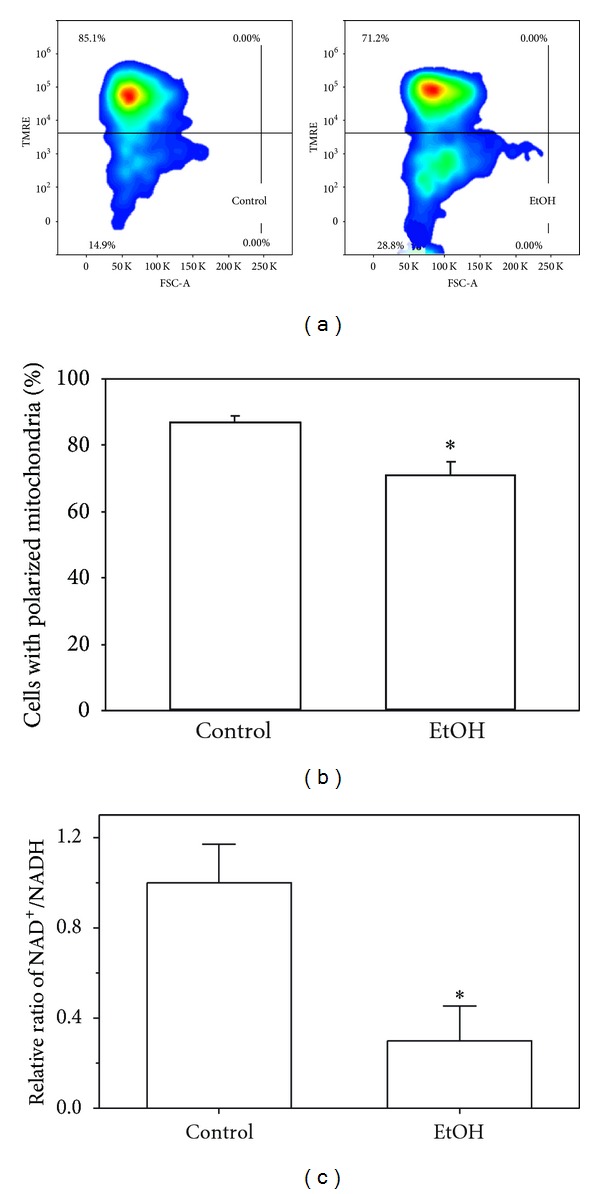
Chronic EtOH exposure promoted mitochondrial depolarization and dysfunction in AMs. mAMs were collected from control and EtOH-fed mice (*n* = 4) and mitochondrial membrane potential was analyzed by FACS analysis of TMRE staining. Representative contour plots of mAMs stained with TMRE showed that chronic EtOH ingestion increased the AM population with lower TMRE staining (a). The quantification of the percentage of mAMs maintaining higher TMRE fluorescence intensity (polarized mitochondria) (b). The ratios of NAD^+^/NADH in isolated mAM mitochondria were determined by using colorimetric assay ((c); pooled AMs, *n* = 5). All values are expressed as mean ± SD and normalized to untreated or control conditions. *P < 0.05 for EtOH *versus* control.

**Figure 4:**
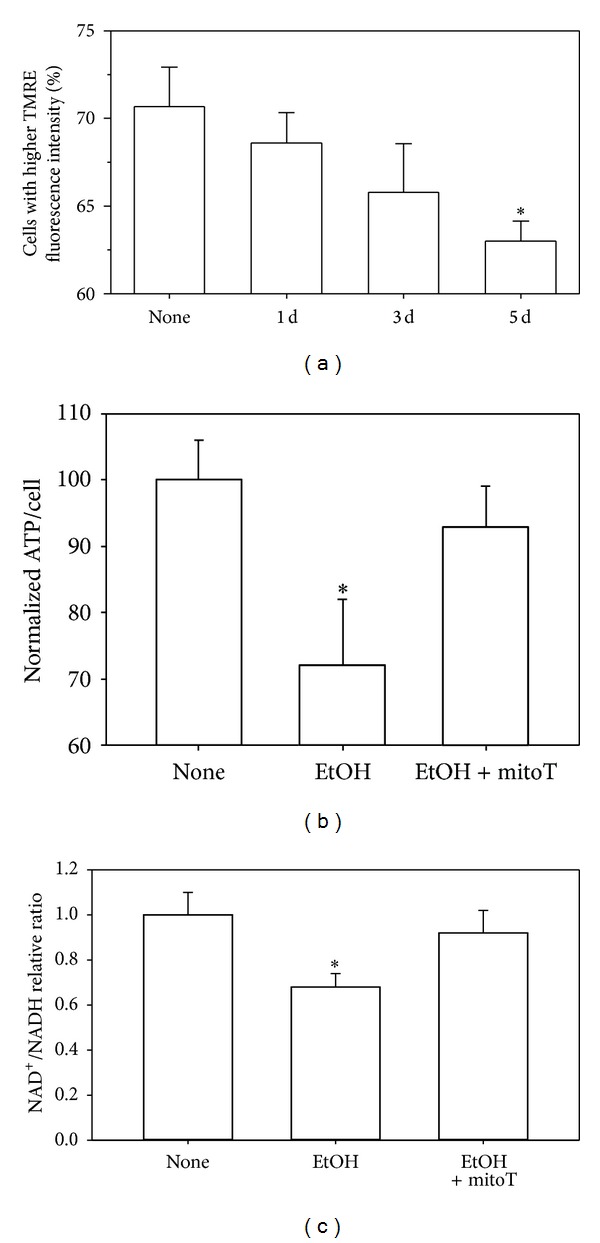
Chronic EtOH exposure promoted mitochondrial depolarization and dysfunction in MH-S cells. MH-S cells were exposed to EtOH for 1d, 3d, and 5d (0.2%, *n* = 3). Following ethanol exposure, MH-S cells were stained with TMRE for FACS analysis (a). In addition to ethanol, some MH-S cells were treated with mitoT for 5d before the ATP (b) or NAD^+^/NADH (c) levels were determined (*n* = 6). All values are expressed as mean ± SD and normalized to untreated conditions. *P < 0.05 for EtOH *versus* none.

Mitochondrial membrane potential is a key indicator of mitochondrial function and integrity. It can be determined with TMRE, a cell permeable cationic dye that readily accumulates in active mitochondria because of the relative negative charge of the mitochondrial membrane potential. EtOH exposure resulted in two mitochondrial populations with TMRE staining. The population with higher TMRE intensity represents those with polarized mitochondria and greater capacity to transport the fluorophore. The population with lower TMRE intensity represents the depolarized mitochondria and decreased capacity to transport the fluorophore. In mAMs, chronic EtOH ingestion decreased the population of cells with higher TMRE staining by 10% (Figures 3(a) and 3(b)) suggesting loss of mitochondrial membrane potential. Similarly, EtOH exposure of MH-S cells incrementally decreased the percentage of cells with higher TMRE staining relative to the period of EtOH exposure (Figure 4(a)). In addition, *in vitro* and *in vivo* alcohol exposure also decreased the ratio of NAD^+^/NADH (Figures 4(c) and 3(c)). Since NADH is oxidized to NAD^+^ in the process of transferring electrons in the mitochondrial electron transfer chain, decreases in the NAD^+^/NADH ratio indicate a mitochondrial redox imbalance and loss of mitochondrial function. Indeed, this EtOH-induced loss of mitochondrial membrane integrity and perturbations in the NAD^+^/NADH ratio were accompanied by a ~25% decrease in ATP production (Figure 4(b)). Ethanol-induced decreases in ATP and NAD^+^/NADH levels were both normalized through the addition of mitoT (Figures 4(b) and 4(c)).

### 3.3. Chronic EtOH Exposure Induced Mitochondria Condensation and Perinuclear Clustering

**Figure 5:**
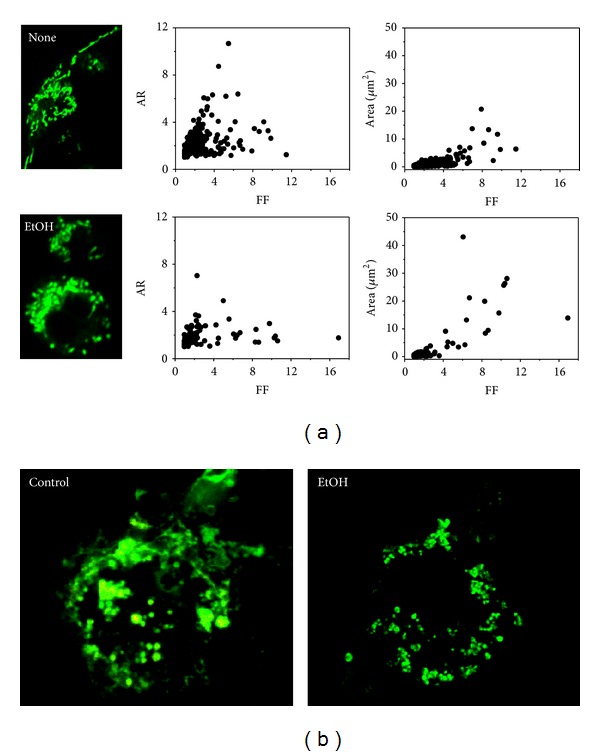
EtOH exposure induced mitochondrial fragmentation, condensation, and perinuclear clustering. Mitochondrial morphology analysis was monitored using mitoTracker green. Representative confocal microscopic images for untreated (none) and EtOH treated (0.2%, 5d) MH-S cells are shown in (a). The value of form factor (FF) and aspect ratio (AR) was plotted for each treatment condition (*n* > 5). For identification of mitochondria, mAMs from control or EtOH-fed mice were loaded with mitoTracker green (representative confocal microscopic images in (b)).

Mitochondria are dynamic organelles which constantly change their size and shape by fusion and fission and their morphological dynamics are linked to the regulation of normal cell physiology and disease. We next examined whether the EtOH-induced mitochondrial ROS generation and mitochondrial depolarization were linked to changes in mitochondrial morphology. In control MH-S, the mitochondrial network was spread throughout the cell (Figure 5(a)). With 5 consecutive days of EtOH exposure (0.2%), the majority of MH-S cells had mitochondria that were condensed and located in the perinuclear region (Figure 5(a)). To quantitatively address mitochondrial morphology changes, we analyzed mitochondrial morphology using a computer-assisted morphometric analysis, which calculates form factor (FF) and aspect ratio (AR) as discussed above. With a minimal value of 1 representing a perfect circle (major axis = minor axis), the mitochondria within control MH-S cells had AR values distributed above 4 suggesting that the mitochondria were elongated. With EtOH exposure, the majority of the AR values were below 4, suggesting a transition from an elongated shape to a more spherical shape. In addition, mitochondrial areas exceeded 15 *μ*m^2^ in the EtOH treatment group suggesting mitochondrial clustering. We next investigated the mitochondrial morphology after chronic EtOH ingestion.  Figure 5(b) is comprised of representative confocal microscopic images of AMs isolated from control and EtOH-fed mice. Because primary mAM cells were taken from their original environment in mouse lungs, they are more fragile, and their morphology was not well retained like that for the MH-S cell line. However, the mitochondria in the AMs from the EtOH-fed mice were more fragmented and clustered at the perinuclear area when compared to the AMs from the control mice.

### 3.4. EtOH-Induced Impairment of Macrophage Phagocytosis Was Reversed by mitoT

**Figure 6:**
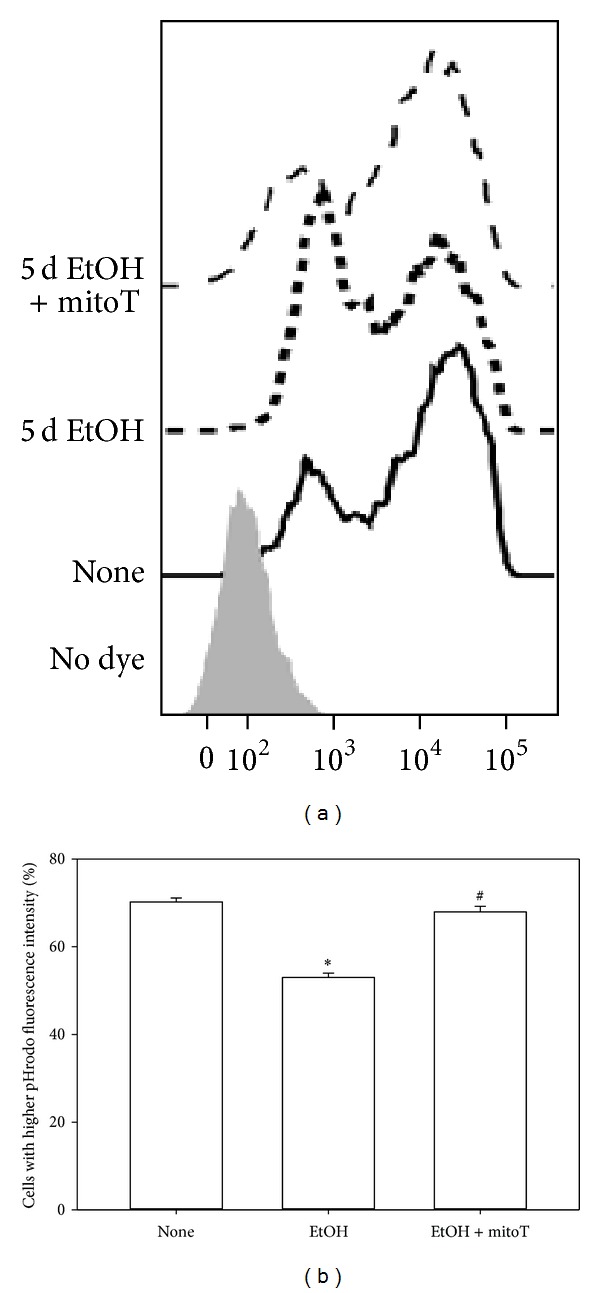
EtOH disrupted AM phagocytosis. Cultured MH-S cells were either untreated (none) or EtOH treated (5d, 0.2%, *n* = 3). During the last 24 hr of EtOH treatment, mitoT was also added to some cells. Cells were then incubated with pHrodo Red *S. aureus *bioparticles conjugate for 2 hr before FACS analysis. Flow cytometry histogram of MH-S cells treated with *S. aureus *bioparticles conjugate uptake (a). Mean fluorescence intensity of pHrodo Red *S. aureus *bioparticles conjugate at each treatment condition (b). All values are expressed as mean ± SD and normalized to the untreated condition. *P < 0.05 for EtOH *versus* none and ^#^ *P* < 0.05 for EtOH *versus* EtOH + mitoT.

**Figure 7:**
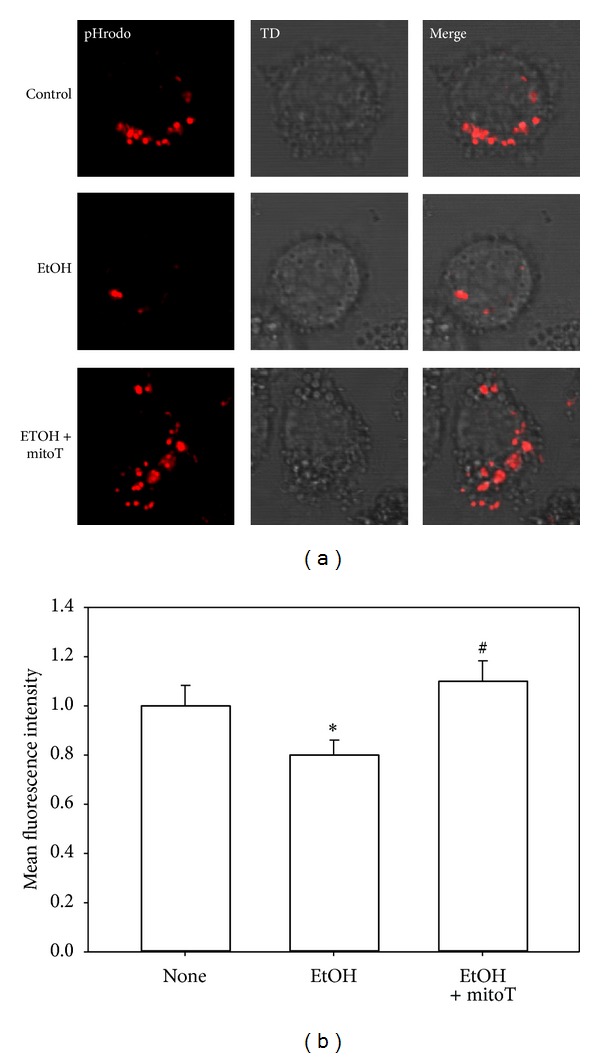
Live cell imaging of mAMs phagocytosis. mAMs were collected from control and EtOH-fed mice, treated *in vitro* with mitoT for 24 hrs, and then incubated with pHrodo Red *S. aureus* bioparticles conjugate for 2 hr. Representative images are shown in (a). The fluorescence mean intensity of pHrodo red per cell was quantified (b). All values are expressed as mean ± SE and normalized to control conditions. *P < 0.05 for EtOH *versus* control and ^#^ *P* < 0.05 for 5d EtOH *versus* EtOH + mitoT.

As demonstrated previously [9, 10], EtOH exposure decreased the phagocytic capacity of mAMs and MH-S cells. Since mitoT attenuated cellular and mitochondrial ROS, we next examined whether mitoT would reverse the effects of ethanol on phagocytosis. As demonstrated in our previous studies, EtOH exposure suppressed phagocytosis of the *S. aureus* bioparticle conjugates by 30% (Figure 6). Treatment with mitoT during the last 24 h of EtOH exposure restored the phagocytic ability of MH-S cells suggesting that mitochondrial-derived oxidative stress was central to EtOH-induced disruptions in phagocytosis. We also examined whether mitoT could restore phagocytosis to mAMs from ethanol-fed mice. Similar to that observed with MH-S cells, *in vitro* treatments with mitoT restored phagocytosis to the mAMs suppressed by chronic ethanol ingestion (Figure 7). These results further confirmed the association between EtOH-induced mitochondrial oxidative stress and impaired mAM phagocytosis.

### 3.5. EtOH Induced Early Apoptosis but Was Prevented by mitoT Treatment

**Figure 8:**
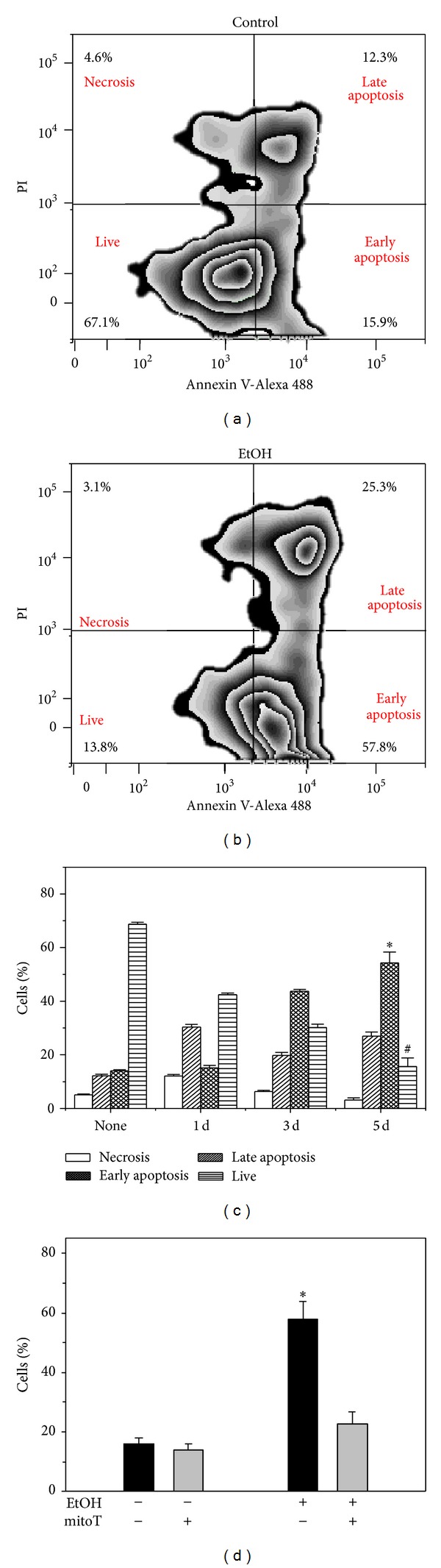
EtOH-induced apoptosis was blocked by mitoT. Cultured MH-S cells were either untreated (none) or EtOH treated (1, 3, or 5d, 0.2%, *n* = 3). Cells were then incubated with the Dead Cell Apoptosis Kit with Annexin V Alexa Fluor 488 and Propidium Iodide (PI) (Invitrogen, Carlsbad, CA) before being analyzed by flow cytometry. A representative flow image for MH-S cells without EtOH treatment is shown in (a). A representative flow image for MH-S cells treated with EtOH for 5 days is shown in (b). The fluorescence mean intensity for live, early apoptotic (Annexin V positive), late apoptotic (positive for Annexin V and cytosolic shrinkage), and necrotic cells (positive for propidium iodide) is shown in (c). During the last 24 hr of EtOH treatment, mitoT was also added to some MH-S cells before assessment of Annexin V staining (d). All values are expressed as mean ± SD and normalized to the untreated condition (*n* = 4). For (c), * denotes *P* < 0.05 for EtOH *versus* none and ^#^ denotes *P* < 0.05 for EtOH *versus* EtOH + mitoT. For (d), * denotes *P* < 0.05 compared to the no treatment group.

As demonstrated previously [9, 10], chronic ethanol ingestion increases AM apoptosis 3-fold with ~30% of the cells expressing markers of apoptosis. In MH-S cells with 5 days of ethanol exposure, the percentage of cells with an early marker of apoptosis, Annexin V positive staining, increased 5-fold (Figures 8(a)–8(c)) when compared to the control group. For cells positive for Annexin V plus loss of cytoplasm, ethanol increased the percentage of cells positive for late apoptosis but statistical significance was not achieved. There also was no statistically significant increase in the percentage of cells positive for a marker of necrosis, propidium iodide staining of DNA (Figure 8(c)). When MH-S cells were pretreated with mitoT, ethanol-induced early apoptosis, Annexin V positive staining, was attenuated (Figure 8(d)).

## 4. Discussion

Chronic alcohol abuse is associated with an increased risk of respiratory infections, pneumonia, and tuberculosis, even in those without a clinical diagnosis of an alcohol use disorder [1, 3]. However, the underlying mechanisms by which alcohol abuse increases the risk of respiratory infections are unclear. One central effect of chronic alcohol ingestion is severe oxidative stress and depletion of critical antioxidants [7]. Within the alveolar space, GSH levels in the alcoholic subjects were significantly decreased when compared with those of nonalcoholic subjects. In the alveolar epithelial lining fluid, alcohol abuse caused an ~40 mV change in the glutathione and glutathione disulfide (GSH/GSSG) redox potential (Eh) [6, 7, 34]. Changes in the extracellular GSH/GSSG redox status were echoed in the intracellular GSH/GSSG redox balance of alveolar type II cells. Indeed, chronic alcohol ingestion caused a 60% decrease in GSH and induced GSH/GSSG oxidation by 40 mV in alveolar type II cells from ethanol-fed adult male rats [35]. In the mitochondria of type II cells, chronic alcohol ingestion also induced a 60 mV oxidation of the GSH/GSSG redox potential when compared to the cells from control rats [35]. For AMs, the GSH/GSSG redox state was oxidized by ~30 mV after chronic ethanol ingestion [36]. Across intracellular and extracellular GSH pools in alveolar cells, the GSH/GSSG redox state was consistently oxidized by 30–60 mV. AMs are the only intra-alveolar phagocyte that responds to inflammation [37] and their function is dependent on the oxidation/reduction balance in the alveolar lining fluid. Indeed, *ex vivo* GSH antioxidant supplementation can reverse the EtOH-induced suppression of phagocytosis in rodent models of chronic alcohol abuse [36].

Previous studies in our laboratory demonstrated that EtOH promotes oxidative stress in AMs through increased ROS production by NADPH oxidases (Nox) [10]. In that mouse model, chronic EtOH ingestion increased the level of mRNA and protein expression of Nox1, Nox2, and Nox4. Since mitochondria produce 90% of cellular ROS compared to a 10% cytosolic ROS contribution under baseline conditions [38], we examined the potential contribution of mitochondria to the ROS. Like the alveolar type II cells [35], the current study demonstrated that EtOH exposure (*in vitro* or *in vivo*) also upregulated mitochondrial ROS generation. Under baseline conditions, increased mitochondrial superoxide production can be enzymatically dismutated to hydrogen peroxide which is subsequently removed by catalase or glutathione peroxidase. However, chronic increases in mitochondrial ROS generation can overwhelm normal scavenging mechanisms, promote the uncoupling of the respiratory chain, and result in more ROS generation, loss of mitochondrial membrane potential, and decreased ATP. In the current study, a role for ethanol-induced mitochondrial ROS was further demonstrated by treatment of AM cells with mitoTEMPOL (mitoT) which reversed EtOH-induced mitochondrial ROS. MitoT contains a lipophilic triphenyl-phosphonium cation added to the TEMPOL antioxidant moiety which promotes its accumulation in the mitochondria. The TEMPOL moiety is a piperidine nitroxide, which has been widely used as a mitochondrial specific antioxidant for *in vivo* and *in vitro* studies. The TEMPOL moiety has superoxide dismutase activity that promotes the detoxification of ferrous iron and prevents toxic hydroxyl radicals formation in the reaction of H_2_O_2_ with ferrous iron [39, 40]. Although our analysis of ROS by redox sensitive fluorophores has its limitations, treatment with mitoT blocked or reversed EtOH-induced ROS generation in the mitochondria further supporting that EtOH promoted mitochondrial ROS. Whether EtOH-induced mitochondrial ROS is due to interference with mitochondrial redox balances [41] or other mechanisms remains to be determined. Furthermore, mitoT also blocked the ethanol-induced increases in cytosolic ROS suggesting that mitochondrial ROS contributes to the generation of cytosolic ROS. Additional studies are needed to determine whether ROS generation through NADPH oxidases, CYP2E1, or other ROS generators are dependent on mitochondrial ROS.

EtOH-induced mitochondrial ROS was also associated with mitochondrial dysfunction. Mitochondrial integrity and function were interrupted by EtOH as evidenced by decreased mitochondrial membrane potential in MH-S cells. More importantly, mAMs from EtOH-fed mice displayed decreased mitochondrial function as evidenced by decreased mitochondrial membrane potential and ATP. The ratio of NAD^+^/NADH was also decreased in both the *in vitro* and *in vivo* models of ethanol exposure. Although the isolation protocol may have increased NAD^+^ and NADH leak from the mitochondria, it is unlikely that the leak of one component would be preferential over the other. Since the actual leak may differ between mitochondrial preparations, we decided that expressing the concentrations of the two components as a ratio would be more accurate. In these studies, NAD^+^ was 0.33 ± 0.03 a.u. (absolute unit) and NADH was 0.20 ± 0.01 a.u. for the control group. For the ethanol group, NAD^+^ was 0.25 ± 0.03 a.u. and NADH was 0.76 ± 0.20 a.u. Therefore, the decrease in NAD^+^ and increase in NADH resulted in a decrease in the mitochondrial NAD^+^/NADH ratio suggesting its oxidation in the mitochondria.

Mitochondria are organelles which supply energy for normal cellular functions making it a key regulator of cell function. For the AM, the energy intensive cellular process such as phagocytosis is particularly dependent on the capacity of the mitochondria to generate ATP. In the current studies, EtOH exposure promoted significant mitochondrial morphological changes, a central indicator of the organelle's integrity and function. In the control group, there was a network of mitochondria with an elongated shape. With EtOH exposure, the mitochondria became more spherical in shape and were present in condensed perinuclear clusters. These EtOH-induced mitochondrial morphological changes are generally associated with cellular oxidative stress and are associated with cell death [35]. As with ethanol-induced mitochondrial ROS, treatment with mitoT normalized mitochondrial NAD^+^/NADH as well as the ATP pool after ethanol exposure. This further supports a causative role for ethanol-induced mitochondrial ROS in the corresponding mitochondrial dysfunction. As observed in previous studies with ethanol-fed animals [36], there was impaired phagocytosis, a key immune function of AMs. However, *in vitro* mitoT treatments reversed the injurious effects of EtOH on the mitochondria and restored the phagocytic capacity even in mAMs from mice fed ethanol for 12 weeks. In addition to impaired bacterial clearance, ethanol decreased cell viability as evidenced by increased early markers of apoptosis. The capacity of mitoT treatment to block apoptosis suggested a central role for ethanol-induced mitochondrial dysfunction in the apoptotic process.

Since mitoT decreased ethanol-induced mitochondrial- and cytosolic-derived ROS, one potential mechanism for the beneficial effects of mitoT could be through its positive cytosolic effects. In previous studies, we demonstrated that chronic EtOH ingestion increases the production of TGF-*β* and IL-13 in AMs which subsequently promotes alternative activation (M2 activation) [7]. In that study, TGF-*β* and IL-13 activated an autocrine loop that was central to AM alternative activation. EtOH-induced upregulation of TGF-*β* expression also promoted another self-activating autocrine loop with the constitutively active NOX 4 resulting in chronic cytosolic ROS generation [10]. Additional studies are needed to determine whether the ethanol-induced activation of TGF-*β*/IL-13 and the TGF*β*/NOX 4 autocrine loops are causative or secondary to ethanol-induced mitochondrial dysfunction. Alternatively, the primary driver could be through ethanol-induced mitochondrial ROS that overwhelm the large antioxidant capacity of the mitochondria, leak into the cytosol, and activate various mechanisms for cytosolic ROS such as the TGF-*β*/NOX 4 autocrine loop. In the current study, mitoT attenuated ethanol-induced mitochondrial dysfunction such as ROS, decreased ATP levels, and decreased NAD^+^/NADH. The mechanisms by which mitoT maintains these events that are critical for the highly energy-dependent processes of phagocytosis and maintenance of cell viability are unclear but may be through maintenance of mitochondrial GSH/GSSG, a critical event for type II cells [35].

Although each piece of data is not singularly definitive, the collective data from diverse measures indicated that ethanol increased mitochondrial ROS: (1) increased cellular ROS as indicated by CM-H2DCFDA oxidation which was blocked by the mitochondrial specific antioxidant mitoT; (2) increased mitochondrial ROS as indicated by MitoSOX fluorescence which was also blocked by mitoT; (3) increased cellular and mitochondrial ROS with *in vitro* and *in vivo* EtOH exposure; (4) the ability of mitoT to attenuate cellular and mitochondrial ROS in the AMs even after chronic EtOH ingestion; and (5) increased oxidation of the mitochondrial NAD^+^/NADH ratio. This mitochondrial oxidation was also associated with mitochondrial dysfunction as evidenced by loss of mitochondrial morphology, depolarization of the mitochondrial membrane potential, and decreased ATP generation. The mitochondria-targeted antioxidant mitoT not only reversed EtOH-induced mitochondrial and cytosolic ROS generation, it also reversed EtOH-induced mitochondrial dysfunction, restored AM phagocytosis, and maintained cell viability. Phagocytosis and cell viability are both complex cellular processes that are controlled at multiple points; additional studies are needed to determine the actual roles of mitochondrial ROS in EtOH-induced disruption of these cellular events. Chronically, alcohol can dampen the inflammatory responses of alveolar macrophages and the chronic suppression of phagocytosis decreases the capacity of alveolar macrophages to clear microbes. Therefore, EtOH-induced mitochondrial ROS and dysfunction in AMs may be pivotal in the increased risk of respiratory infections and ARDS in subjects with an alcohol use disorder.
